# Prevention in Thermal Crack Formation in Chabazite (CHA) Zeolite Membrane by Developing Thin Top Zeolite and Thick Intermediate Layers

**DOI:** 10.3390/nano11082113

**Published:** 2021-08-19

**Authors:** Min-Zy Kim, Syed Fakhar Alam, Devipriyanka Arepalli, Aafaq ur Rehman, Won-Youl Choi, Churl-Hee Cho

**Affiliations:** 1 Graduate School of Energy Science and Technology, Chungnam National University, 99 Daehak-ro, Yuseong-gu, Daejeon 34134, Korea; mzkim@cnu.ac.kr (M.-Z.K.); fakhar9689@o.cnu.ac.kr (S.F.A.); devipriya0508@o.cnu.ac.kr (D.A.); aafaqktk@cnu.ac.kr (A.u.R.); 2 Department of Advanced Materials Engineering, Gangneug-Wonju National University, Gangneung 25457, Gangwon, Korea

**Keywords:** chabazite zeolite membrane, nanosize seed, seed penetration, intermediate layer, themal crack, pervaporation

## Abstract

Chabazite (CHA) zeolite membranes with an intermediate layer of various thicknesses were prepared using planetary-milled seeds with an average particle diameter of 300, 250, 200, 140, and 120 nm. The 120 nm seed sample also contained several smaller particles with a diameter of 20 nm. Such small seeds deeply penetrated into the pore channels of the α-alumina support during the vacuum-assisted infiltration process. During the secondary growth, the penetrated seeds formed a thick intermediate layer exiting between the zeolite layer and support. A decrease in seed size increased the penetration depth of seeds and the thickness of the intermediate layer, while the thickness of seed coating and zeolite layers was decreased. CHA zeolite membranes with a thin top zeoliate layer and a thick intermediate layer showed an excellent water/ethanol separation factor (>10,000) for 90 wt.% ethanol at 70 ℃ with a total flux of 1.5 kg m^−2^ h^−1^. There was no observation of thermal cracks/defects on the zeolite separation layer. The thick intermediate layer effectively suppressed the formation of thermal cracks during heating, since the tensile stress induced in the zeolite layer was well compensated by the compressive stress on the support. Therefore, it was successfully proven that controlling the microstructure of top surface and intermediate layers is an effective approach to improve the thermal stability of the CHA zeolite membrane.

## 1. Introduction

The dehydration of organic solvents using a pervaporation membrane has caught extreme attention due to its low energy consumption and environment-friendly nature [1,2]. Compared to other membrane types, the zeolite membrane is one of the most promising dehydration membranes due to its high dehydration performance [3,4,5]. In recent years, hydrophilic and acid-resistant zeolite membranes such as MER [6], MOR [7], ZSM-5 (MFI) [8], T (OFF) [9] and CHA [10]-type with a Si/Al molar ratio of 2–10 were reported for dehydration of organic mixture in acidic conditions. Among them, the CHA-type zeolite membrane was studied extensively in recent years.

CHA-type zeolite framework is a 3-dimensional interconnected microporous system with an accessible 3.8 × 3.8 Å window of small eight-membered rings and large ellipsoidal 6.7 × 10 Å cavities [11]. Pure-silica (Si-CHA) [12], aluminosilicate (chabazite [13] and SSZ-13 [14]) and silicoaluminophosphate (SAPO-34) [15] belong to the CHA-type zeolite. The chabazite zeolite with a Si/Al molar ratio of 2 to 5 shows a higher acid-resistance in solvent dehydration compared to the commercially available NaA zeolite membrane with a Si/Al molar ratio of 1 [16,17,18,19]. Hence, CHA zeolite membranes with a high Si/Al molar ratio are considered as prominent candidates for water separation from an organic solvent mixture in an acidic environment.

CHA zeolite has a large negative thermal expansion (NTE) coefficient, a maximum of −16.7 × 10^−6^ K^−1^ over the temperature range of 20–600 °C, as reported by Woodcock et al. [20]. Usually, zeolite membranes are synthesized on porous supports (such as alumina, mullite, and stainless steel) that provide sufficient mechanical strength and have a positive thermal expansion (PTE) behavior. If there is a mismatch in the thermal expansion coefficient between the support and the zeolite layer, the induced tensile stress in the zeolite layer is exposed during heating, which leads to forming grain boundaries or trans-granular cracks [21]. Therefore, it is important to prepare a CHA zeolite membrane capable of resisting the formation of these structural defects. 

Recently, the CHA zeolite membranes with a Si/Al ratio of 2–5 were synthesized by a secondary growth process using Sr^2+^, K^+^, and Na^+^ cations as mineralizing agents, in the absence of organic structure-directing agents (OSDA) [22,23,24,25]. The exclusion of OSDA is preferred because it does not require an energy-intensive calcination process. However, introducing only cations tends to result in a weak interaction framework structure compared to using OSDA. Furthermore, cations form a zeolite framework through interaction with negatively charged aluminum-occupied oxygen tetrahedral ([AlO_2_]^−^) in the zeolite lattice. Therefore, the cations could provide proximity to the zeolite framework when the Al content is high [26,27,28]. Noack et al. suggested that high aluminum present in the synthesis solution could affect the growth of defect-free MFI membranes because the surface charge of the zeolite framework becomes more negative [29]. However, these requirements are not sufficiently satisfied with cations when the silica content of zeolite increases [26]. Increasing the silica content in the zeolite leads to weak interactions in the framework and forms defects. Therefore, it is difficult to resist the difference in the coefficient of thermal expansion (CTE) between the zeolite layer and the support. In the literature related to the preparation of the CHA zeolite membrane with Si/Al with ratio 2–5, thermal defects are easily observed in the microstructures [16,17,18,22,23,30,31,32,33]. Most of the membranes showed a separation factor of about 2000 to 4000 for 90 wt.% ethanol-water mixture at 75 °C [13,16,23,31,32,33]. Further, the reported CHA membranes having a high separation factor (>10,000) show interfacial defects, which could affect long-term stability [18]. Therefore, to prevent the thermal crack, and structural defects formation in the CHA zeolite membrane, it is necessary to mitigate the difference in CTE of the zeolite layer and support. 

The utilization of porous support with a low CTE can be an important strategy to improve the thermal stability of zeolite membranes. However, the use of lower CTE mullite support does not show much resistance to defects in the microstructure and no significant improvement in the performance was observed [13,32]. 

In our earlier reports, it was proved that the thermal stability of the NaA zeolite membrane was improved by introducing the well-developed intermediate layer in which the NaA zeolite and support phases were three-dimensionally interconnected with each other [34]. The nanosize seeds penetrated the pore channels of the support with a relatively large pore size of 0.65 µm during a vacuum infiltration process. In the secondary growth process, penetrated seeds grew outward along the pore channels and formed a thermally stable intermediate layer with a thickness of 6–26 µm. The tensile stress induced in the zeolite layer was compensated by the compressive stress on the support. 

In this present study, defect-free CHA zeolite membranes with a thick intermediate layer were synthesized to improve thermal stability and dehydration performance. The intermediate layer was formed during the secondary growth by using nanosize seeds, which penetrated the internal pore of the α-alumina support. For this, a simple and useful top-down ball-mediated milling process was adopted. Various nanosize seeds were prepared by adjusting the planetary milling conditions such as milling time and speed. The effect of the seed size on the formation and pervaporation performance of CHA zeolite membranes was extensively investigated.

## 2. Experimental

### 2.1. Preparation of CHA Zeolite Membranes

Nanosize seeds were prepared by a high energy planetary ball-miller (Pulverisette 6 Planetary Mono Mill, Fritsch, Germany) using lab-synthesized K-CHA zeolite microspheres with a diameter of 5 µm and 0.5 mm zirconia balls. The synthesis process and growth mechanism were already reported [35]. K-CHA zeolite particles of 5 g and zirconia balls of 40 g were placed in a milling jar containing 140 mL of distilled water. The K-CHA zeolite particles were planetary-milled at different rotation speeds of 200 to 400 rpm for varying durations of 6–100 h, respectively. In the present study, five planetary milled seeds (S1 to S5) were prepared. After milling, each seed was re-dispersed in distilled water without drying to prepare a colloidal seed solution of 0.005 wt.% and stored in a PP bottle after ultrasonication. The detailed milling conditions are summarized in Table 1.

CHA zeolite membranes were synthesized by the secondary growth process on the surface of single-channel porous α-alumina tubular support. The α-alumina supports were prepared by the slip-casting method [36]. The length and outer diameter of support were 200 and 8 mm, respectively. The milled S1–S5 seeds were coated on the outer surface of the α-alumina support by a vacuum-assisted infiltration process [37]. The seeded α-alumina supports were dried at 100 °C for 24 h before the secondary growth. 

Five different membranes (M1–M5) were synthesized by secondary growth on the seeded support (S1–S5). For the secondary growth, the synthesis solution was prepared by dissolving colloidal silica (LUDOX^®^ HS-40, 40 wt.% suspensions in water, Sigma-Aldrich, St. Louis, MO, USA), aluminum nitrate (≥98%, Sigma-Aldrich, St. Louis, MO, USA), strontium nitrate (97%, Junsei Chemicals, Tokyo, Japan) and potassium hydroxide (≥85%, pellets, Sigma-Aldrich, St. Louis, MO, USA). Firstly, the aluminate solution was made by mixing the potassium hydroxide and aluminium nitrate in de-ionised water. Secondly, strontium nitrate was mixed with the de-ionised water, and then colloidal silica was added slowly to the strontium nitrate aqueous solution in order to make a silicate solution. Later on, the as-made aluminate solution was added dropwise into the silicate solution and stirred for 6 h at room temperature. The molar composition of the hydrothermal solution was 12SiO_2_:Al_2_O_3_:2K_2_O:SrO:8KNO_3_:780H_2_O [17]. The resultant molar gel solution was poured into a Teflon-lined stainless-steel autoclave containing seeded support and then the synthesis was carried out at 140 °C for 24 h. Finally, prepared CHA zeolite membranes were washed with de-ionised water until the pH of washing water reached about 7 and finally dried at 100 °C overnight. 

The pore characteristics of the α-alumina support were analyzed by mercury porosimeter (Thermo Scientific PASCAL 440, Milan, Italy). Particle size distributions of the original zeolite and milled seeds were measured by dynamic light scattering (DLS, Microtrac-Nanotrac wave-particle size analyzer, Microtrac MRB, Haan, Germany) at room temperature. Powder X-ray diffraction (XRD) studies of seeds, the α-alumina support and the synthesized CHA zeolite membranes were made on a PANalytical: X’Pert PRO diffractometer (Panalytical, Almelo, Netherlands) with Cu- Kα radiation (*λ* = 1.5418 Å) at 40 kV and 100 mA. The data was collected in a 2θ range of 10–60° with a step size of 0.03° s^−1^. The XRD patterns were indexed and phase identification was performed with the help of JCPDS file #98-003-2553. Scanning electron microscopy (SEM) images were collected by using a field emission scanning electron microscope (JEOL JSM-7000F, Tokyo, Japan). During the morphological studies on SEM, the elemental composition was measured with energy dispersive spectrometry (EDS).

### 2.2. Characterization of Pervaporative Ethanol Dehydration and Single Gas Permeation Properties

The solvent dehydration performance of prepared CHA zeolite membranes was characterized by pervaporation (PV) for a water-ethanol mixture [37]. A prepared CHA zeolite membrane was sealed in a membrane module. In the PV test system, 90 wt.% water-ethanol mixture of 1 L was poured into the feed tank and heated up to 70 °C with a flow rate of 1L/min. The permeate side was evacuated using a vacuum pump and collected with liquid nitrogen traps at each time interval. The permeate flux was calculated by permeate mass and membrane area (47 cm^−2^) at each time interval, and the H_2_O/EtOH separation factor (SF) was determined by TCD gas chromatography (GC) measurement of the chemical compositions obtained at the feed and permeate sides at each time interval. The permeation flux was calculated as follows:

*J* = *w*/(*A* × *t*) (1)

where *w* is the weight of the collected permeate vapor, *A* is the area of the membrane, and *t* is the collecting time. The separation factor was calculated from the formula below:

*α_water/ethanol_* = (*Y_w_*/*Y_e_*)/(*X_w_*/*X_e_*) (2)

where *Y_w_/Y_e_* is the weight ratio of water to ethanol in the permeate and *X_w_/X_e_* is the weight ratio of water to ethanol in the feed.

Single-gas permeation experiments were carried out for He, CO_2_, O_2_, N_2_, and SF_6_ gases at the absolute feed pressure of 3 bar and the atmospheric permeate pressure, i.e. a Δ*p* of 2 bar. The flow rate was controlled to 1000 mL/min by mass flow controllers. Before the single gas permeation test, the prepared membranes were cleaned by He sweeping at 100 °C for 12 h. The gas flux was measured with a bubble flow meter (1–250 mL/min a bubble generator, Gilibrator 2, Gilian, USA) and applied for the single gas permeance. The permselectivity was calculated by using single gas permeances.

## 3. Results and Discussion

### 3.1. Milling and Coating of Nanosize Seeds

**Figure 1:**
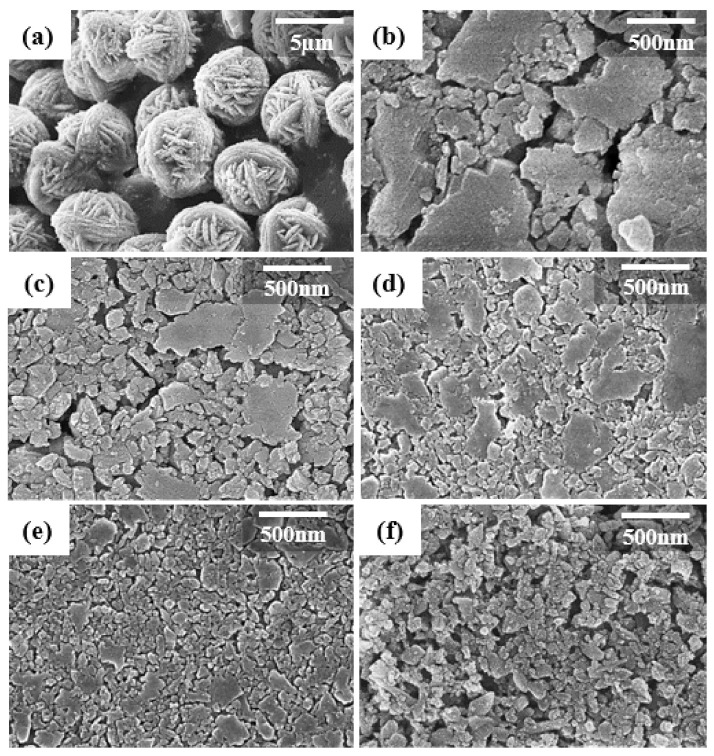
SEM images of (**a**) original CHA zeolite particles and planetary-milled seeds with different milling speed and time; (**b**) S1 = 200 rpm for 6 h, (**c**) S2 = 200 rpm for 12 h, (**d**) S3 = 200 rpm for 24 h, (**e**) S4 = 400 rpm for 36 h, and (**f**) S5 = 400 rpm for 100 h.

The SEM images for the original CHA zeolite particles and planetary-milled S1–S5 seeds prepared at different milling conditions are shown in Figure 1. 

The CHA zeolite particles before milling were thread ball-shape intergrown microspheres with a diameter of 5 µm (Figure 1a), which were composed of truncated bi-pyramidal primary particles [35]. The S1–S3 seeds were planetary-milled at 200 rpm for 6, 12, and 24 h. During the milling process, the micro spherical shape particles were converted to irregularly shaped particles with different particle sizes as displayed in (Figure 1b–d). Both irregular large particles of size range between 400 to 600 nm and small particles of size less than 200 nm were typically observed. The seeds S4 and S5 milled at 36 and 100 h at 400 rpm were ground into relatively smaller and uniform particles of size about 100 nm are seen in (Figure 1e,f). The small particles were slightly agglomerated and had a rounded surface. Also, it is expected that small and rounded particles were activated due to the high surface energy due to the small size and the mechanical deformation during the milling. It is interesting to know how such an activated seed can affect the growth of zeolite crystals during secondary growth process.

**Figure 2:**
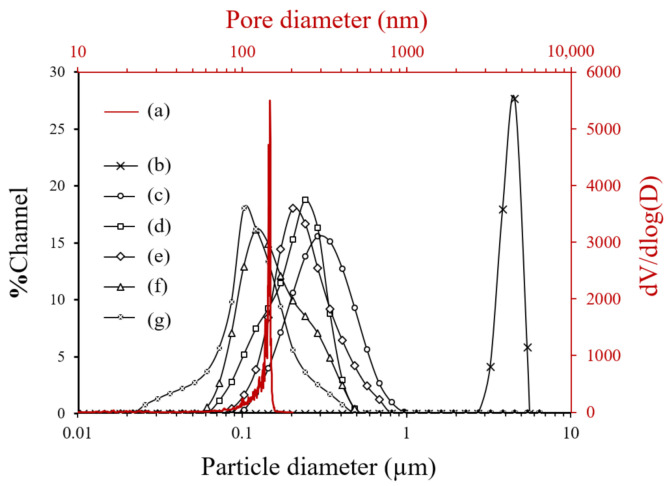
(**a**) Pore size distribution of the porous α-alumina support and particle size distribution of (**b**) original CHA zeolite particles and (**c**–**g**) planetary-milled S1 to S5 seeds.

Figure 2 exhibits the particle size distribution of the original CHA zeolite particles and milled seeds of S1 to S5 and the pore size distribution of α-alumina support. 

The pore size distribution of the α-alumina support shows a very narrow single peak at around 150 nm (Figure 2a). The average pore size and porosity were 120 nm and 35%, respectively. The particle size distribution of original CHA zeolite particles was observed with a single peak at a range of 4–5 µm (Figure 2b). The planetary-milled seeds with different milling conditions show a wide range of broad peaks and shifted to the left of the x-axis with increasing milling speed and time (Figure 2c–g). The DLS seed size data were well consistent with the microstructure results shown in Figure 1. Notably, in the S4 and S5 seeds planetary-milled at 400 rpm for 36 and 100 h, the mean size was smaller than the pore size of the α-alumina support. In the case of S5, the smallest size measured by DLS was decreased to around 20 nm, much smaller than the pore diameter of the support. It was expected that a few tens nm small particles in the S4 and S5 seeds penetrate the support surface to the inside of the support during the vacuum infiltration seeding. Table 1 includes the range and average particle size of seeds according to the milling conditions. 

The S1–S5 seeds had an average diameter of 300, 250, 200, 140, and 120 nm, respectively. However, the milled seeds contain a large number of nanosize particles, smaller than the pore diameter of supports. The lower limit of particle size decreased as the milling speed and time increased, as listed in Table 1.

**Figure 3:**
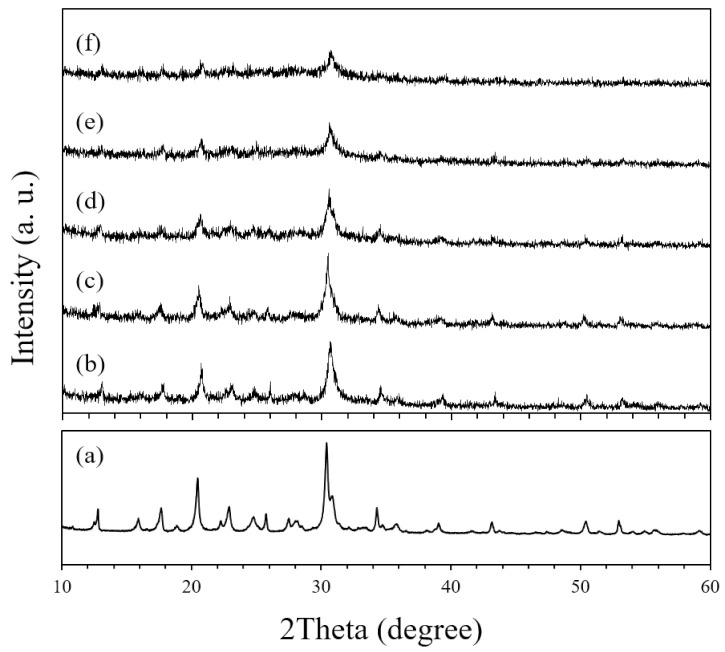
X-ray diffraction patterns of (**a**) original CHA zeolite particles and (**b**–**f**) planetary-milled S1 to S5 seeds.

Figure 3 shows the X-ray diffraction patterns of original CHA zeolite particles and planetary-milled S1–S5 seeds. 

The original CHA zeolite has high phase purity and high crystallinity (Figure 3a). In all XRD patterns of seeds, only CHA zeolite peaks were found (Figure 3b–f), while the intensities of crystalline peaks diminished with extending milling speed and time. It is clear that in addition to the particle size, the crystallinity of CHA zeolite particles decreased during the milling. During the milling, the particles were severely deformed as shown in Figure 1, and also it is expected that the plastically deformed seeds are activated, so that can make an effect on the secondary growth of the zeolite layer.

**Figure 4:**
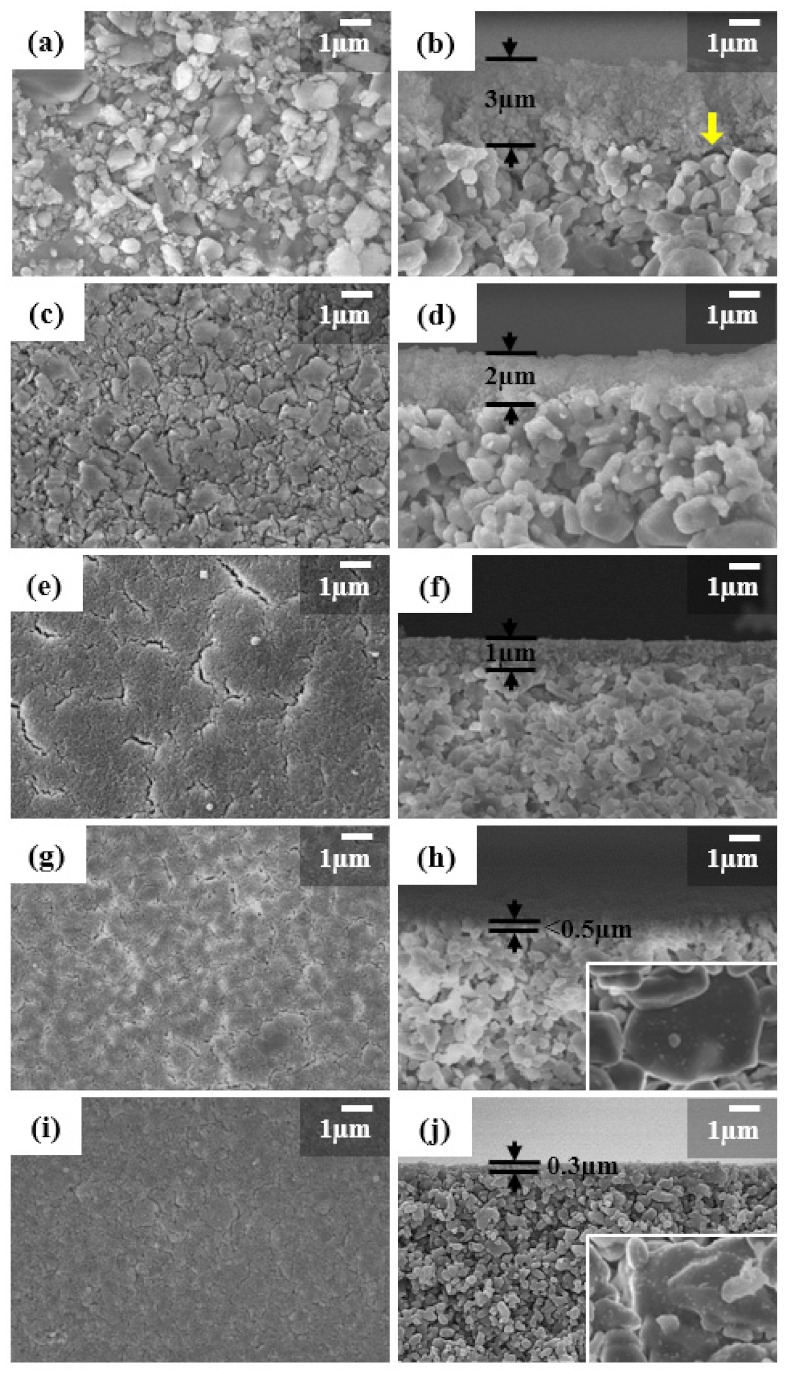
Surface and cross-section SEM images of seeded supports using planetary-milled seeds with average particle size of (**a**,**b**) S1 = 300 nm, (**c**,**d**) S2 = 250 nm, (**e**,**f**) S3 = 200 nm, (**g**,**h**) S4 = 140 nm and (**i**,**j**) S5 = 120 nm. The yellow arrow shows detachment of seed layer.

The effect of seed size on the coating of the seed layer was investigated. The vacuum-assisted infiltration coating method was applied to introduce the milled seeds on the outer surface of the support. Figure 4 shows SEM images for the top-surfaces and cross-sections of seeded supports. 

The seeds uniformly covered the entire surface of supports and formed a seed coating layer, with different thicknesses and densities, highly dependent on the seed size. The smaller seeds made a more compact seed layer with higher coverage. As the seed size decreased from S1 to S5 the thickness of the seed coating layer was significantly decreased from 3 to 0.3 µm. A rough seed coated layer and some detachment in seed layers were observed when the S1 seeds with an average size of 300 nm were used to coat the supports as shown by arrows in Figure 4b. In the S2 and S3 seeds, the seed coating layer became flattened and well adhered to the support surface (Figure 4c–f). However, cracks were formed on the surface of seed layers during drying. Finally, the S4 and S5 seed layers were uniform and dense (Figure 4g–j). The cracks formed during drying became smaller and smaller with decreasing seed size. Finally, it was difficult to find cracks in the S5 seed layer. In addition, small seeds of a few tens nm penetrated the support surface and can be observed in the interior of support, as shown in the insets of Figure 4h,j.

### 3.2. Synthesis of CHA Zeolite Membrane 

**Figure 5:**
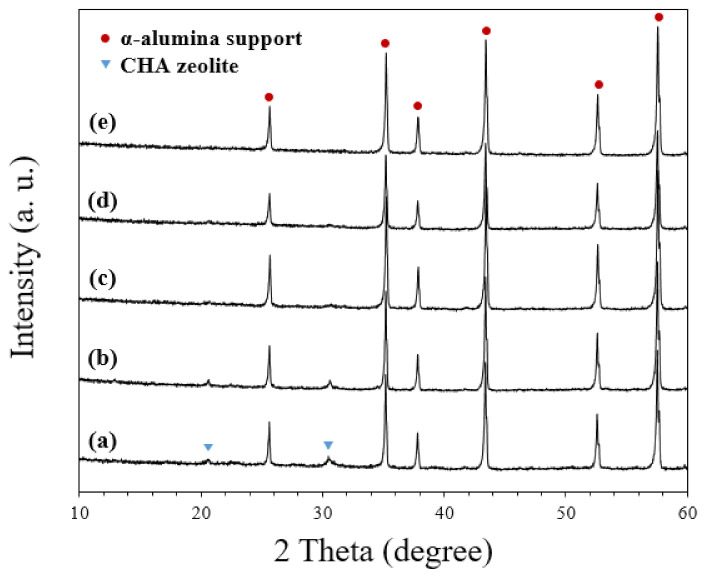
X-ray diffraction patterns of synthesized CHA zeolite membranes using planetary-milled (**a**) S1, (**b**) S2, (**c**) S3, (**d**) S4, and (**e**) S5 seed samples.

CHA zeolite membranes were prepared by the secondary growth using S1–S5 seeds and the effect of seed size on the formation of zeolite and intermediate layers was investigated. Figure 5 shows X-ray diffraction patterns of membranes prepared by using various seeds. 

**Figure 6:**
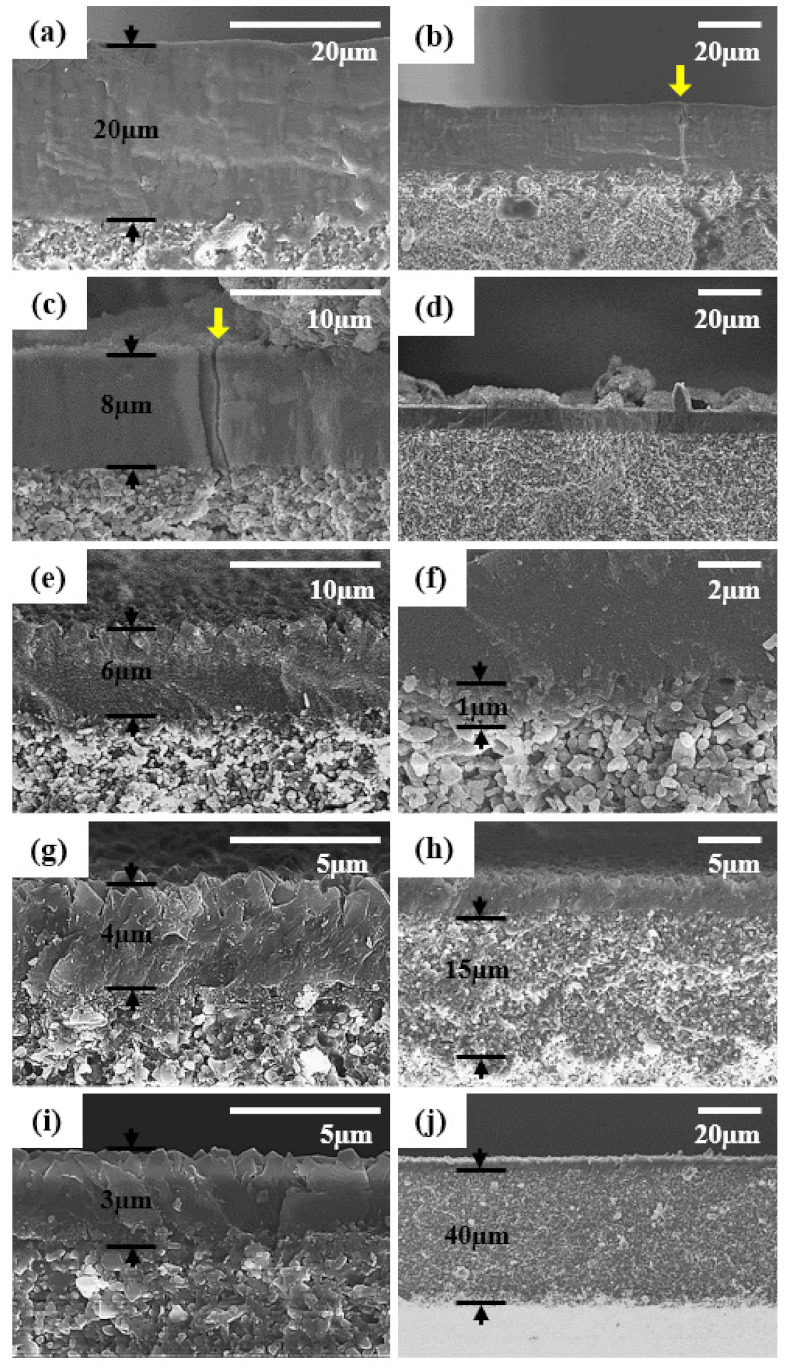
Cross-sectional SEM images of synthesized CHA zeolite membranes using planetary-milled (**a**,**b**) S1, (**c**,**d**) S2, (**e**,**f**) S3, (**g**,**h**) S4, and (**i**,**j**) S5 seed samples. The yellow arrow shows site of cracks in the zeolite membrane.

High-intensity alumina characteristic peaks were displayed in all the samples. The intensities of CHA zeolite characteristic peaks were relatively weak and were directly related to the amount of CHA zeolite phase present in the X-ray penetration depth and the ability of diffracted beams to escape. The escape ability will increase when the diffracted beam is closer to the top surface. Considering that X-ray penetration depth is a few hundred µm, the zeolite phase is present in the top zeolite layer and the intermediate layer is in the X-ray diffraction sphere. As shown later in SEM images (Figure 6), as the seed size decreases, the thickness of top CHA zeolite layer decreases from 8 to 3 µm. Therefore, the weak intensities of the XRD peak in the membranes synthesized using smaller seeds were mainly from the small thickness of the top CHA zeolite layers; the small thickness of the top CHA zeolite layers in those membranes were induced from the small thickness of seed layers as shown in Figure 4.

The cross-sectional SEM images of CHA zeolite membranes prepared by changing seeds are presented in Figure 6. All the SEM images were taken after the PV tests. As the seed size decreased, the thickness of the CHA zeolite membranes reduced from 20 to 3 µm. The CHA zeolite membranes synthesized using the S3 to S5 seeds had an intermediate layer. In the intermediate layers, zeolite crystals were formed between the alumina grains of support (Figure 6f,h,j). The thickness of the intermediate layer increased to 1, 15, and 40 µm, as the seed size decreased. There was no thermal crack observed in SEM images of the CHA zeolite membranes with intermediate layers. On the contrary, there was no intermediate layer in the CHA zeolite membranes synthesized using the relatively large seeds of 300 and 250 nm, and even the cross-sectional SEM images confirmed the thermal cracks in the zeolite layer (highlighted by the arrows in Figure 6b,c).

**Figure 7:**
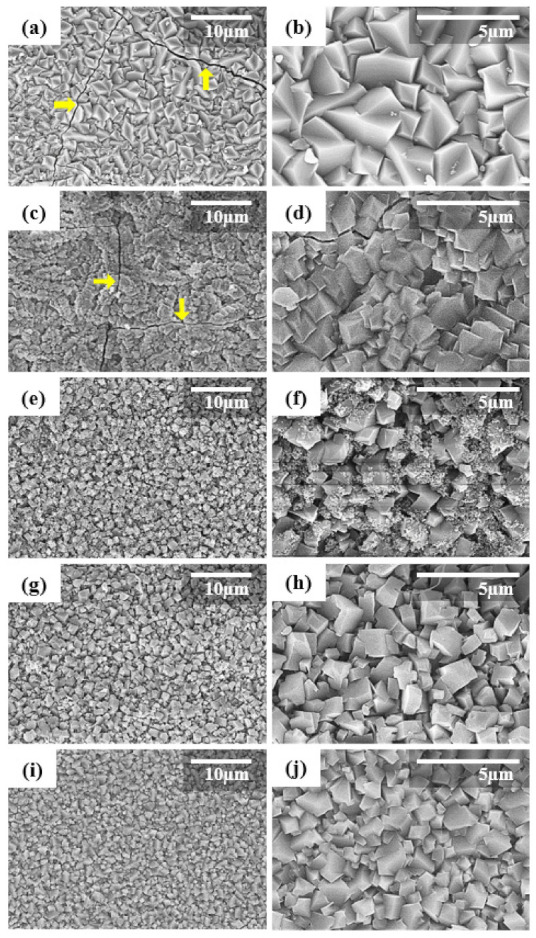
Different magnification top-surface SEM images of the synthesized CHA zeolite membranes using planetary-milled (**a**,**b**) S1, (**c**,**d**) S2, (**e**,**f**) S3, (**g**,**h**) S4, and (**i**,**j**) S5 seed samples. The yellow arrow shows site of cracks in the zeolite membrane.

Figure 7 shows low and high magnification SEM images for the top-surfaces of the synthesized membranes. 

In all the membranes, truncated bipyramid shape crystals, which is the typical shape of CHA zeolite crystals, covered the full surface of the support. As the seed size decreased, the bipyramidal grains became more uniform and their size decreased from 4 µm to approximately 1 µm. Especially, as the seed size decreased, it became more difficult to find cracks on the top surface of the zeolite layer. Large transgranular and sharp cracks were clearly observed in the membranes synthesized using S1 and S2 seeds (arrows in Figure 7a,c). Such cracks are typically thermal cracks, generated by the difference in the coefficient of thermal expansion (CTE) between the zeolite layer and the support [34,37]. Additionally, thermal cracks/structural defects were easily observed in the SEM images of CHA zeolite membranes reported in the literature [13,18,19,23,31,32,33,38]. On the other hand, no cracks were observed in the CHA zeolite membranes synthesized using S3 to S5 seeds (Figure 7e–j), and the microstructure was more uniform. 

**Figure 8:**
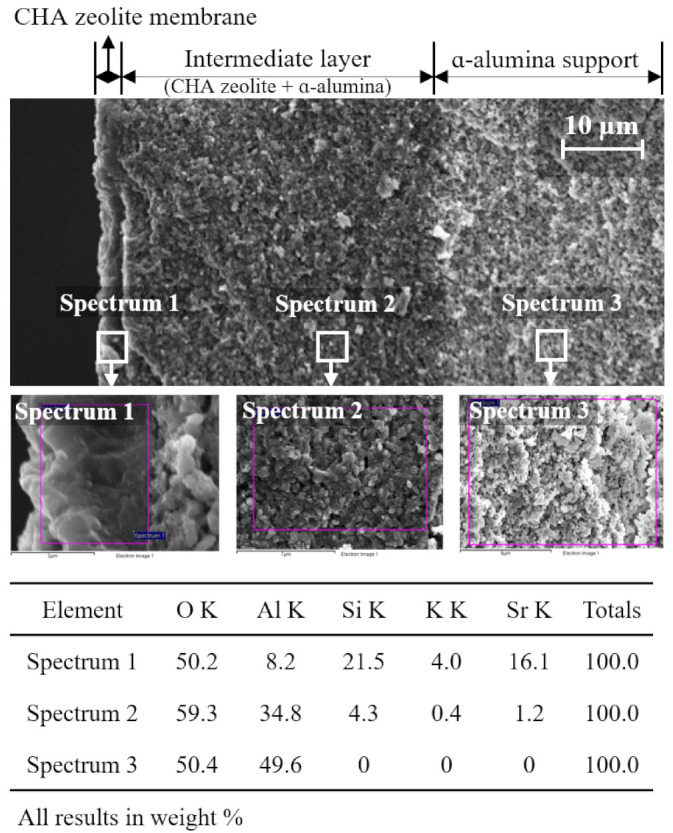
EDS results for three parts of M5 CHA zeolite membrane: (spectrum 1) zeolite separation layer, (spectrum 2) intermediate layer, and (spectrum 3) support.

EDS analysis was performed to identify the intermediate layer of the membrane prepared by using the S5 seed. Figure 8 shows EDS results for the three parts belonging to the zeolite layer, the intermediate layer, and the alumina support. 

In the zeolite layer (spectrum 1), Al, Si, O, K, and Sr elements are detected, and the elements are typical components of zeolite crystals. The Si/Al molar ratio of the zeolite layer was 2.6. In the support (spectrum 3), only Al and O elements were observed. On the other hand, in the intermediate layer (spectrum 2), a high wt.% of the Al element and low wt.% of Si, K, and Sr elements were identified. Accordingly, it was confirmed that the pore of the support was filled with well-grown zeolite crystals as shown in the SEM images of Figure 8. 

Other synthesis parameters influencing the formation of the intermediate layer are crystallization time and the SiO_2_/Al_2_O_3_ ratio of hydrothermal solution [13,39]. Liu et al. reported that the increase of crystallization time in the synthesis of the LTA zeolite membrane effectively improved crystal intergrowth and developed the intermediate layer [39]. Their study also announced that the membrane with a thick intermediate layer of ~5 µm showed long-term stability compared to conventional membranes for a 50 wt.% aqueous methanol solution at 60 ℃ for one week. Jiang et al. synthesized a well-intergrown CHA zeolite membrane in a broad SiO_2_/Al_2_O_3_ ratio range and compared the pervaporation performance [13]. From their microscopic images, it was confirmed that the thick intermediate layer of ~10 µm was formed as the SiO_2_/Al_2_O_3_ ratio increased to 10–15. Their result was probably due to the aluminum ions being partially dissolved from the support surface due to the high pH of the hydrothermal solution [40]. In the present study, since the same synthesis solution and synthesis time were applied for the 5 membranes, it was concluded that the intermediate layer was developed by changing the ratio of seed size to pore diameter of support.

**Figure 9:**
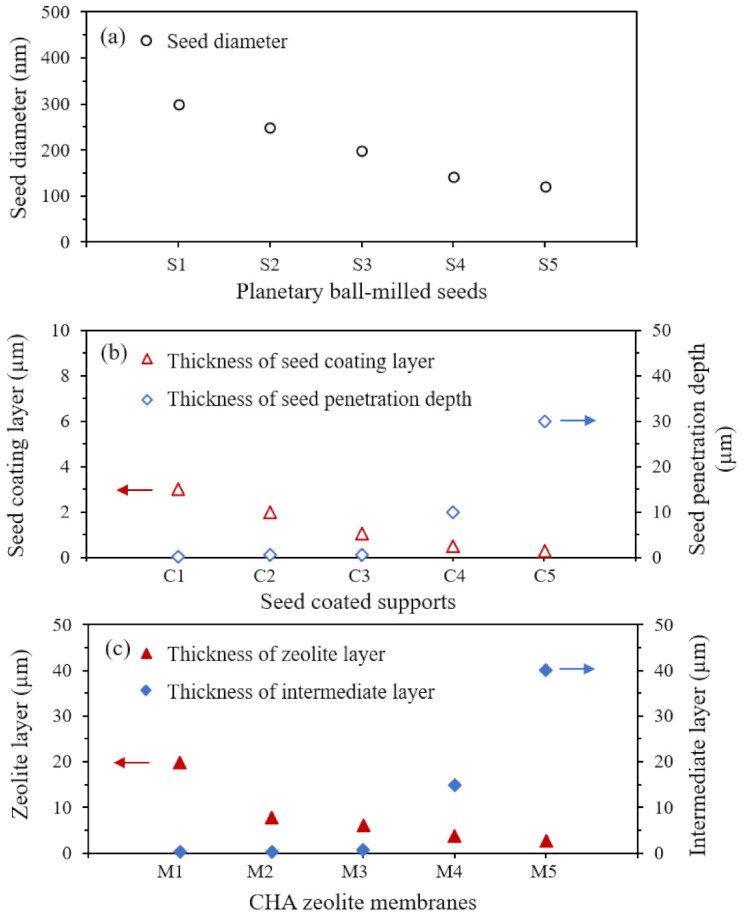
Summary on the variations of (**a**) average seed diameter, (**b**) thickness of seed coating layer and seed penetration depth, and (**c**) thickness of CHA zeolite layer and thickness of the intermediate layer.

Figure 9 summarizes variations in the thickness of the seed coating layer, the seed penetration depth, the thickness of the zeolite layer, and the thickness of the intermediate layer as a function of seed size. 

**Figure 10:**
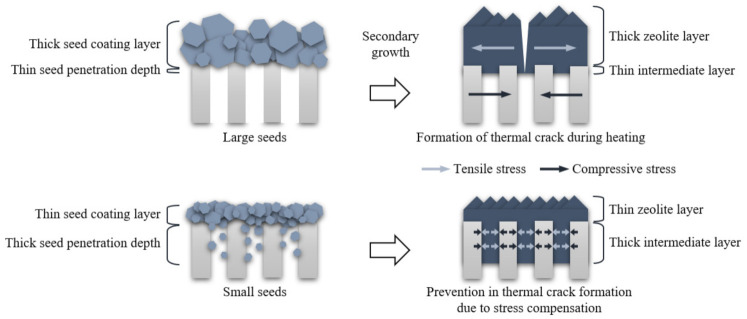
The schematic diagram for the formation of CHA zeolite membrane and prevention in thermal crack formation by controlling seed size.

As the seed size decreased, the seed penetration depth and the thickness of the intermediate layer increased, while the thickness of the seed coating layer and the thickness of the zeolite layer decreased. The reason why small seeds develop a thick intermediate layer and improve the thermal stability of zeolite membrane was systematically investigated by our group for the NaA zeolite system [34]. As nanosize seeds penetrate pore channels of support during the seeding process, the growth of the nanosize seeds in the pore channels during the secondary growth process induces a thick intermediate layer. The thick intermediate layer increases the thermal stability due to compensating tensile stress in the zeolite layer with compressive stress on the support during heating. In the present study, the same phenomenon is demonstrated in the CHA zeolite system, which is schematically represented in Figure 10. 

Therefore, it was clearly elucidated that the development of the intermediate layer by controlling the seed size relative to the pore diameter of the support is an important approach to improve the thermal stability of the zeolite membrane. In addition, thin top zeolite layers in the M4 and M5 membraens contributed to the high thermal stabilities.

### 3.3. PV Performance of CHA Zeolite Membranes

**Figure 11:**
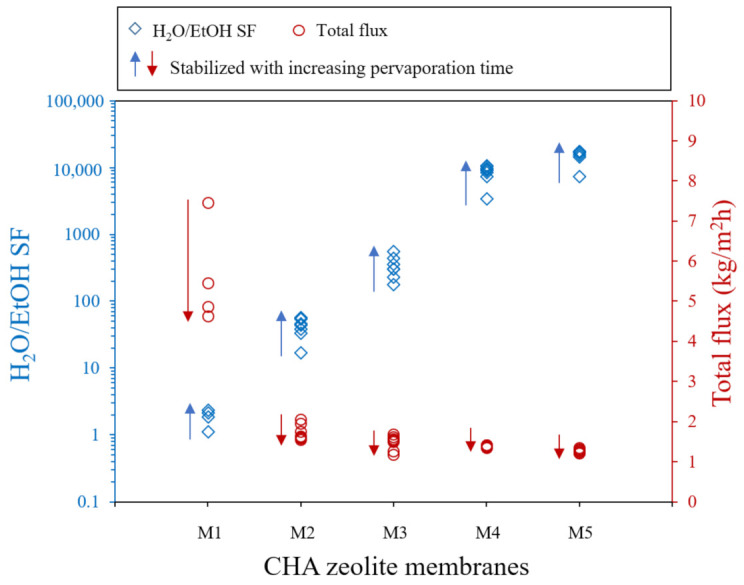
PV performance of synthesized M1-M5 CHA zeolite membranes for 90 wt.% ethanol-water mixture at 70 °C. The arrows indicate increasing pervaporation time.

The thermal crack-free CHA zeolite membranes are expected to show high separation performance. Therefore, the pervaporation performance of M1–M5 membranes was evaluated for 90 wt.% ethanol-water mixture at 70 °C. The H_2_O/EtOH separation factor and total flux were calculated by sampling feed and permeate every hour in the pervaporation test. The results are shown in Figure 11.

As the pervaporation proceeded, the separation factor gradually increased, the flux decreased and the PV performance of each membrane showed stable performance within a few hours. With increasing the intermediate layer thickness, the total flux and separation factor were more rapidly stabilized. The M1 membranes without an intermediate layer show a low water/ethanol separation factor and a high total flux due to thermal cracks observed in the zeolite separation layer (Figure 6a). As mentioned previously, such thermal cracks were generally observed in the CHA zeolite membranes reported in many references [13,18,19,23,31,32,33,38]. The M4 and M5 CHA zeolite membranes that had a thick intermediate layer showed a high water/ethanol separation factor of more than 10,000 and a stable total flux of about 1.5 kg m^−2^ h^−1^. The low flux and high separation factor of M4 and M5 membranes mean formation of a continuous zeolite layer: there was no thermal crack and the permeation resistance was increased by the thick intermediate layer. Additionally, the S4 and S5 seeds with high strain and surface energies might accelerate the growth of zeolite crystals due to the enhanced solubility [41].

Table 2 summarizes the PV performances of CHA zeolite membranes for 90 wt.% ethanol-water mixture reported in literature and synthesized in the present study.

An attempt was made to compare the formation of the intermediate layer from the SEM images, and the relationship between the seed size and the pore diameter of support was investigated. Interestingly, in the case of using a seed smaller than the pores of the support, an intermediate layer of more than 5 µm was formed in most cases [13,23,24,31]. It shows that the intermediate layer grown with the aid of small seeds made a significant effect on the PV performance. In addition to the intermediate layer, the selection of the support materials was important. However, the use of high CTE support such as stainless steel support (10.1–17.3 × 10^−6^ K^−1^) and YSZ (10.5 × 10^−6^ K^−1^) was not effective in preventing thermal defects even though the intermediate layer was well developed [13,16,18,24,31,33,42]. In their SEM images, an interfacial gap was observed between the zeolite layer and the support and most membranes showed thousands of separation factors. In addition, mullite support with relatively low CTE (5 × 10^−6^ K^−1^) was applied and formed a thick intermediate layer [31,40]. However, defects due to the non-uniformity and impurity of the zeolite crystal existed, which was caused by the formation of an impurity MER zeolite phase. On the other hand, according to the present study, it is clear that using alumina support (7.2 × 10^−6^ K^−1^) with an intermediate layer can improve both thermal stability and PV performance significantly. However, the total flux of as-synthesized CHA zeolite membrane in this study was relatively lower than with the literature. The main mass transfer resistance in pervaporation using zeolite membrane is located in the supports [43]. This is because the support properties such as porosity, tortuosity, pore size and thickness affect the total mass transfer in the membrane [44].

Single gas permeation performance was tested to check the continuity of the CHA zeolite layer. Table 3 shows single gas permeation test results for He, N_2_, O_2_, CO_2,_ and SF_6_. 

Even though gas permeances gradually decreased with decreasing of the seed size, the permeances of SF_6_ with a kinetic diameter of 5.5 Å for the M4 and M5 membranes were still very high, around 0.61–0.71 × 10^−7^ mol m^−2^ s^−1^ Pa^−1^. Also, the He/SF_6_ permselectivity is 4.15, which is lower than the Knudsen selectivity (6.04). These results announce that the membranes showing almost infinite H_2_O/EtOH separation factors still contain a large number of non-zeolitic pores, even though there is no large crack. Nevertheless, water molecules migrate selectively through the membranes. This means that water molecules selectively permeate through the zeolitic and non-zeolitic pores present in the membrane. The selective water permeation is because water molecules condense in zeolitic and nonzeolitic pores under the PV condition [37,45]. The non-zeolitic pores fill with condensed water molecules like liquid water. The selective condensation of water in zeolite and non-zeolite pores prevents organic solvents from entering the pores, resulting in high water selectivity. Finally, when a vacuum is applied on the permeate side, the condensate can evaporate into the permeate side.

From the PV performance and single gas permeation data, it was clearly shown that the CHA zeolite membrane having an intermediate layer showed an excellent PV performance because there was no crack and wide non-zeolitic pores in the membrane. 

## 4. Conclusions

CHA zeolite membranes were prepared by varying seed size. The average seed size was controlled to be 120 to 300 nm by changing planetary milling conditions. The smallest seed size was around 20 nm in diameter. Such a small nanosize seed effectively penetrated the pore channel of the support and remained a thin top seed layer during vacuum assisted seeding. As the average seed size decreased, the seed penetration depth increased and thickness of the top seed layer decreased. The penetrated seeds grew into zeolite crystals in the pores of the support during the secondary growth, remaining an intermediate layer. As the seed penetration depth increased, the thickness of the intermediate layer increased. In the membranes with an intermediate layer of 15–40 µm and a top zeolite layer of 3–4 µm, there was no thermal crack on the zeolite layers. The thin top zeolite and thick intermediate layers effectively prevented the formation of thermal cracks during heating, since the tensile stress induced on the zeolite phase can be well compensated by the compressive stress on the support. Thermal crack-free CHA zeolite membrane showed a high water/ethanol separation factor of more than 10,000 for 90 wt.% ethanol at 70 °C with the total flux of 1.5 kg m^−2^ h^−1^. Therefore, it could be concluded that controlling the microstructure of the top surface layer and intermediate layer is an effective approach to improve the thermal stability of the CHA zeolite membrane.

## Figures and Tables

**Table 1:** Particles size distribution of planetary-milled seeds at various conditions.

Seeds	Ball-Milling Condition		Particle Size	
	Speed (rpm)	Time (h)	Range (nm)	Average (nm)
S1	200	6	100–1000	300
S2	200	12	90–800	250
S3	200	24	70–500	200
S4	400	36	60–500	140
S5	400	100	20–400	120

**Table 2:** Summary of the pervaporation performance of CHA zeolite membrane reported in literature and prepared in this work for a 90 wt.% ethanol-water mixture.

Support	Pore Size (µm)	Seed Size (µm)	Thickness of Intermediate Layer ^a^ (µm)	Thickness of Zeolite Layer (µm)	J_total_ (kg m^−2^ h^−1^)	α^W/E^	Ref.
α-Al_2_O_3_ tube	0.15	6	<2	4	4.1	39,500	[38]
α-Al_2_O_3_ tube	0.31	5–10	Not confirmed ^b^	5	14	>10,000	[10]
Mullite tube	1.3	5.5	<2	10	2.2	3900	[32]
Stainless steel tube	1.8	0.5	>5	8	3.7	2100	[31]
Stainless steel tube	1.8	2–4	<2	4	7.3	2000	[16]
Mullite tube	0.6	0.22	>10	10	2.5	2980	[13]
YSZ hollow fiber	1	0.22	>5	12	8	2500	[33]
YSZ hollow fiber	1	0.22	>5	10	13.3	6000	[23]
YSZ hollow fiber	1	1	<2	8	12	>10,000	[18]
α-Al_2_O_3_ hollow fiber	0.5		Not confirmed ^b^	Not confirmed ^b^	4.91	>2000	[19]
Stainless steel tube	1.8	0.5	>5	3	6.25	1950	[24]
α-Al_2_O_3_ tube	0.15	0.3	0.3	20	4.6	2.3	This work
α-Al_2_O_3_ tube	0.15	0.25	0.5	8	2.3	57.3	This work
α-Al_2_O_3_ tube	0.15	0.2	1	6	1.7	552.3	This work
α-Al_2_O_3_ tube	0.15	0.14	15	4	1.4	>10,000	This work
α-Al_2_O_3_ tube	0.15	0.12	40	3	1.2	>10,000	This work

^a^ Observed in the literature as cross-sectional SEM images of the membranes. ^b^ There is no cross-sectional image of the membrane in the literature.

**Table 3:** Single gas permeance data of synthesized CHA zeolite membranes.

Membranes	Permeance × 10^−6^ (mol m^−2^ s^−1^ Pa^−1^)					Selectivity
	He	N_2_	O_2_	CO_2_	SF_6_	He/SF_6_
M1	3.00	2.14	2.05	1.86	1.30	2.27
M2	3.00	1.86	1.74	1.45	1.02	2.90
M3	3.00	1.47	1.35	1.21	0.86	3.45
M4	2.91	1.26	1.18	1.06	0.71	4.10
M5	2.64	1.10	1.05	0.90	0.64	4.15

## Data Availability

Data can be available upon request from the authors.
